# Machine Learning-Based Extraction of Breast Cancer Receptor Status From Bilingual Free-Text Pathology Reports

**DOI:** 10.3389/fdgth.2021.692077

**Published:** 2021-08-17

**Authors:** Antoine Pironet, Hélène A. Poirel, Tim Tambuyzer, Harlinde De Schutter, Lien van Walle, Joris Mattheijssens, Kris Henau, Liesbet Van Eycken, Nancy Van Damme

**Affiliations:** Belgian Cancer Registry, Brussels, Belgium

**Keywords:** machine learning, natural language processing, breast cancer, pathology, receptor status

## Abstract

As part of its core business of gathering population-based information on new cancer diagnoses, the Belgian Cancer Registry receives free-text pathology reports, describing results of (pre-)malignant specimens. These reports are provided by 82 laboratories and written in 2 national languages, Dutch or French. For breast cancer, the reports characterize the status of estrogen receptor, progesterone receptor, and Erb-b2 receptor tyrosine kinase 2. These biomarkers are related with tumor growth and prognosis and are essential to define therapeutic management. The availability of population-scale information about their status in breast cancer patients can therefore be considered crucial to enrich real-world scientific studies and to guide public health policies regarding personalized medicine. The main objective of this study is to expand the data available at the Belgian Cancer Registry by automatically extracting the status of these biomarkers from the pathology reports. Various types of numeric features are computed from over 1,300 manually annotated reports linked to breast tumors diagnosed in 2014. A range of popular machine learning classifiers, such as support vector machines, random forests and logistic regressions, are trained on this data and compared using their *F*_1_ scores on a separate validation set. On a held-out test set, the best performing classifiers achieve *F*_1_ scores ranging from 0.89 to 0.92 for the four classification tasks. The extraction is thus reliable and allows to significantly increase the availability of this valuable information on breast cancer receptor status at a population level.

## Introduction

**Figure 1:**
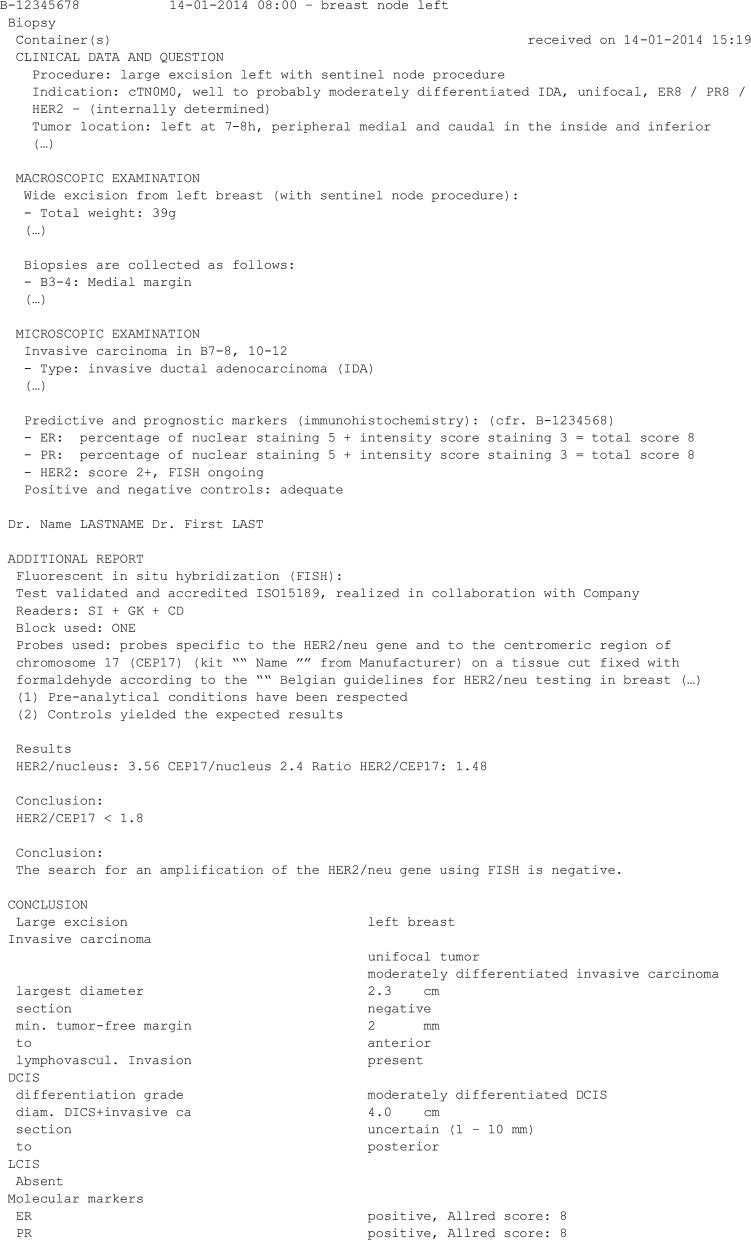
Fictive example of a breast cancer pathology report (translated into English). This report (shortened to only contain the text relevant to the present work) presents results about the status of three biomarkers of interest: ER, PR, and HER2. The status of HER2 can be assessed using immunohistochemistry (IHC) and/or *in-situ* hybridization (ISH).

The Belgian Cancer Registry (BCR) is a population-based cancer registry collecting information on all new cancer diagnoses in Belgium, covering incidences at the population level since 2004 (1, 2). Every oncological care program in the hospitals and every laboratory for pathological anatomy is required by law to register structured information on new (pre-)malignant cases. This structured information covers a range of patient (age, sex…) and tumor (histology, topography, stage…) characteristics. Besides this structured information, pathologists must also provide the free-text reports, written either in Dutch or French, an example of which is displayed in Figure 1. Other examples are available on the BCR website.^1^

In addition to producing statistics on the cancer burden in Belgium (2), the BCR is also involved in several prospective clinical registration projects, the evaluation of quality of care in oncology, the registration of all cyto-histological specimens in the context of screening programs for breast, colorectal and cervical cancer, and in several research projects (http://kankerregister.org/Publications).

Within the context of these activities, having the breast cancer receptor status at its disposal would undoubtedly be of added value. In breast cancer, estrogen receptor (ER), progesterone receptor (PR), and Erb-b2 receptor tyrosine kinase 2 (ERBB2, previously named Human Epidermal Growth Factor 2 or HER2 or HER-2/neu^2^) are biomarkers known to be related to tumor growth and prognosis, and assessing their expression is necessary to define therapeutic management (3–7). Currently this information is not available in a structured form at the BCR. However, the information is usually mentioned in the free-text pathology reports, therefore the BCR is looking for ways to exploit these existing resources instead of demanding additional registration efforts. The goal of the present work is to investigate whether automatic methods could be used to extract ER, PR, and HER2 results out of the pathology reports.

In clinical practice, the analysis strategy is defined by (inter)national guidelines (3, 5, 8, 9). ER and PR expressions are usually tested using immunohistochemistry (IHC) which detects protein expression through immunostaining of the tested fixed tissue (4). The HER2 status is assessed by two complementary methods, IHC and *in situ* hybridization (ISH), which uses DNA probes to detect *HER2* gene amplification (3, 5). At first, the HER2 expression is assessed by semi-quantitative IHC: 0 and 1+ are interpreted as negative, 2+ as equivocal and 3+ as positive. As described by the guidelines, ISH test of HER2 should only be performed in case of equivocal or positive expression of HER2 by IHC. The interpretation of the results including cut-offs for positivity and the categorization of breast cancers in subtypes according to their receptor status have been captured in guidelines (10).

Several natural language processing (NLP) tools have previously been developed to automatically extract ER, PR, and HER2 status from free-text reports written in English (7, 11), Chinese (12), and Bulgarian (6). In general, clinical NLP tools can be separated into three categories, according to the applied methodology: rule-based (7, 13), conventional machine learning (11, 12), and deep learning (6).

Rule-based clinical NLP tools rely on a set of rules written by experts describing how a computer should classify a report. For instance, Dexter et al. wrote a series of rules to identify and classify sentences corresponding information about the biomarker status (7). Designing rule-based tools is very time-consuming, because there are many ways to express the information of interest (6, 11, 12). As an example, Buckley et al. found more than 4,000 different ways of saying that invasive ductal carcinoma of the breast was not present (13).

Conventional machine learning and deep learning NLP tools let the computer create the rules defining how to classify a report. To be able to do so, the computer needs to be provided with many annotated reports, called the training set. Conventional machine learning tools convert words or phrases to high-dimensional numeric vectors indicating whether a word or a sentence appears in the report (11, 12, 14). During this transformation, the sequential order of the sentences in the report is lost. These vectors are given as inputs to conventional machine learning algorithms (such as random forests, support vector machines, logistic regressions, etc.).

Deep learning NLP tools convert words of the report to low-dimensional numeric vectors conveying their meaning, called “embeddings.” The embeddings are sequentially given as inputs to deep learning algorithms (6). Currently, deep learning is the state-of-the-art method for NLP tasks. However, deep learning is computationally intensive, requires a lot of annotated data and the complexity of the resulting models is a potential challenge when it comes to interpretability and explanation to the non-data scientist users of the extracted data.

Inspired by these efforts, the current study presents the first attempt at automatic extraction of ER, PR, and HER2 status from free-text Dutch and French pathology reports in Belgium. The extraction is performed at a national level, involving 82 different data providers (all Belgian laboratories for pathological anatomy). This problem has not been addressed in the previously mentioned studies, which focused on a limited number of data sources (6, 11–13).

## Methods

### Data

Pathology reports processed at the BCR are very heterogeneous, owing to the large number of data providers. Types of heterogeneity include:

- At least two different languages, French and Dutch, sometimes mixed in the same report;
- Different naming conventions and abbreviations. For instance, HER2 is also referred to as ERBB2 (9), Cerb-B2, Her2/neu (3, 5) or Neu, sometimes with additional spaces or dashes;
- Different writing styles: some reports are lists of bullet points, for instance reporting results as “ER: +,” while others are very narrative, for instance “Estrogen receptor stainings are positive”
- Different use of punctuation (sometimes absent), upper case (sometimes only uppercase), and line breaks (sometimes absent).

Because of this large heterogeneity, machine learning tools were anticipated to perform better than rule-based ones.

**Figure 2:**
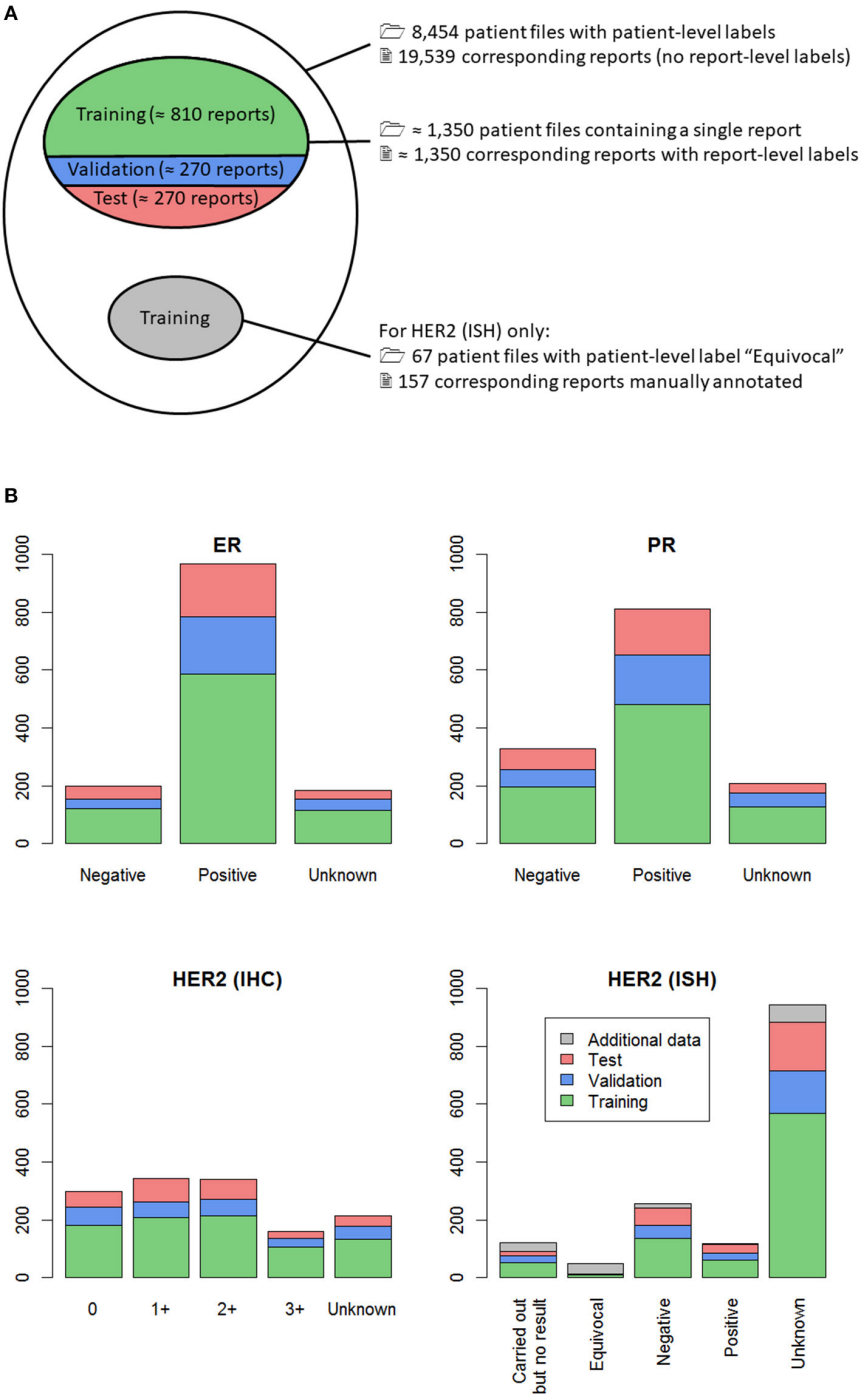
**(A)** Venn diagram of all manually classified patient files (outer ellipse); **(B)** Distribution of the labels in the dataset, for the four classification tasks. **(A)** From all the manually classified patient files, those containing only one report were selected, so that a report-level label was available (upper inner ellipse). This subset of reports was randomly split into training (60%, green), validation (20%, blue) and test (20%, red) sets. For one of the four classification tasks, namely *HER2* (ISH), all patient files with patient-level label “Equivocal” were selected, resulting in 67 patient files, shown in gray. The corresponding 157 reports were individually labeled, and these report-level labels were added to the training set for classification of *HER2* (ISH). **(B)** Split of the data in training (green), validation (blue) and test sets (red) is shown, along with additional classified data for *HER2* (ISH) (gray). For each classification task, training data (green + gray) was used to build several machine learning classifiers, which were evaluated on the separate validation set (blue) to avoid overfitting. Once the best classifier was selected, its performance was assessed on the held-out test set (red).

For the purposes of a larger study, 8,454 patient files of breast cancer patients newly diagnosed in 2014 were manually annotated at the BCR. Each patient file contained one or several reports, written in one of the two national languages, Dutch or French. In total, the 8,454 patient files contained 19,539 reports, as shown in Figure 2A.

The reports contained in each patient file were manually reviewed by one annotator, who gave the file a conclusive label for each biomarker. The annotation work was divided between seven different annotators, trained for this specific task. The biomarkers of interest were given the following possible labels by the annotators:

- ER and PR: “Unknown,” “Negative,” or “Positive;”
- HER2 (IHC) expression: “Unknown,” “0,” “1+,” “2+,” or “3+;”
- *HER2* (ISH) amplification: “Unknown,” “Carried out but no result,” “Negative,” “Equivocal,” or “Positive.”

These sets of possible labels were established with the help of an expert pathologist and according to national and international pathology guidelines (3–5, 8, 9). The labels “Unknown” and “Carried out but no result” are additional informal labels that were used when no test result was mentioned in the report. In the latter case, the available reports state that a test was carried out, unfortunately the test result was not transmitted to the BCR.

The fact that several reports belonging to the same patient can contain contradictory information was anticipated to be a major difficulty. Consequently, the machine learning algorithm development was performed at the report level. Only patients having a single report were selected, as in these cases the link between the patient file label and the report label is unequivocal. This selection resulted in 1,341 to 1,355 reports manually labeled for each of the four classification tasks, as shown in Figure 2A. The minor differences are caused by missing labels, the number of which slightly varied depending on the biomarker. The distribution of the manual labels for all reports is shown in Figure 2B. This figure shows that there were very few reports with manual label “Equivocal” for *HER2* (ISH). To compensate this observed label imbalance, all 67 patient files with manual patient-level label “Equivocal” were selected, and each of the 157 corresponding reports was manually annotated. These additional data are represented in gray in Figure 2.

For each classification task, the set of classified reports was randomly split into training (60%), validation (20%) and test (20%) sets (Figure 2). The training set was used to select the most relevant features and train several machine learning classifiers. Then, the performances of the machine learning classifiers (formally defined in Appendix A) on the validation set were used as a criterion to evaluate and improve them during the development phase. Once the best classifier was selected, its performance was assessed on the held-out test set, allowing to estimate its generalization error.

### Procedure

#### Pre-processing

Each report was first divided into lines, by splitting at each line break. Then, each line was split into sentences, using a home-made sentence tokenizer in the R programming language (15). The reason for splitting into lines and sentences is that some reports do not contain line breaks, while others present results as bullet points, without punctuation (see Figure 1). Finally, each sentence was separated into words using a punctuation-based tokenizer.

#### Feature Engineering

As a next step, more than 20,000 numeric features were extracted from each report. The types of features used were:

- Presence (yes/no) and number of matches for regular expressions (regexes) applied to the whole report and to separate sentences. These regexes were used to detect sequences of words, including negations, and to group synonyms and—as the corpus contains texts in both Dutch and French—different translations of the same word.
- Absolute and relative locations of matches for the previous regexes in the report. Such features are important since the decisive information is very often located at the end of the report, while the beginning typically contains medical history and test indication, which can cause false positives (see Figure 1 for an example).
- Regex-based extraction of usual numeric values related to the possible labels, such as Allred score (provided in Figure 1 for ER and PR) (4), H score (4) and *HER2*/*CEP17* ratio [provided in Figure 1 for *HER2* (ISH)] (3, 5, 8, 9). Such numeric values are not systematically provided in all reports and thus cannot be used as such.
- Word counts, using the tm package in R (16), i.e., the number of times each specific word appears in each document. One such feature that can be useful is the number of times the word “positive” appears in a report, for instance.
- Jaccard similarities between all sentences in a report and selected reference sentences. The Jaccard similarity between two sentences is defined as the ratio between the number of words shared by the two sentences and the total number of unique words in the two sentences. During the error analysis process on the training set, sentences which contained unusual ways of reporting results were identified. Such sentences were then used as reference sentences. For example, an important reference sentence was “CerB2 négatif” (“CerB2 negative;” note the spelling of HER2).
- Combinations of features with the Boolean operators AND and OR. For instance, an important feature was whether “0” occurred more often than “1+,” “2+,” or “3+” in the report.
- Logistic regressions of features.

It was observed that PR test results were very often presented directly after ER test results and followed the same structure, as is the case in Figure 1. Similarly, HER2 (IHC) test results often directly followed PR test results. A methodology was thus developed to detect structure repetition in the reports and to isolate ER, PR, and HER2-related segments of the reports. Feature extraction was performed on the reports as a whole and on these isolated sections, which contributes to the large number of features.

The features used in this work were very heterogeneous and were consequently of different orders of magnitude. This situation can slow down the training of machine learning models and cause the training process to stop too early. To solve this issue, features were normalized. The process is formally defined in Appendix B. The code for feature extraction and the constants for normalization are provided in the Supplementary Materials.

#### Feature Selection

To decrease the number of features in the classifiers and avoid overfitting, coefficients of a regularized multinomial logistic regression were used as a feature ranking method on the training set. This procedure was performed using the function cv.glmnet of the R package glmnet (17).

#### Classifiers and Training

Different classifiers were built on the training set:

- Random forests with a varying number of trees [R package randomForest (18)].
- Support vector machines (SVM) with linear, radial, polynomial and sigmoid kernels [R package e1071 (19)] and different values of the kernel parameters and penalty.
- Regularized multinomial logistic regression [R package glmnet (17)] with different values of the regularization parameter.
- Classification trees, with a maximum depth ranging from 2 to 10 [R package rpart (20)].
- The k-nearest neighbors algorithm [R package class (21)]. The classifier was applied with *k* = 1, 3, 5, 9, 15, 30, and 45 neighbors.

During the training step, self-training (22) was used to try to improve classifier performance. Self-training consists in using a classifier on unlabeled data to artificially generate more training data. In this work, there were more than 18,000 reports without report-level label (Figure 2A, white area inside the largest ellipse). A first classifier was built on the reports having a report-level label and was used to automatically label the remaining reports. The classifier was then re-trained using this larger, artificial training sample. In the specific case of HER2 (IHC), this procedure improved the performance of the classifier, and so, this better classifier was retained.

#### Validation

The macro-averaged *F*_1_ score (11) was used to compare the performance of different classifiers and hyperparameter values on the validation set. The macro-averaged *F*_1_ score is formally defined in Appendix A. This performance metric penalizes error for each label equally. It was chosen as the preferred criterion because of the large label imbalance, as shown in Figure 2B. The macro-averaged *F*_1_ score ranges between 0 and 1, with 0 corresponding to a classifier which is systematically wrong, and 1 corresponding to a perfect classifier.

To estimate baseline performance, four rule-based classifiers were also developed, one for each classification task. Some of the previously developed regexes can be directly linked to a label and were selected for rule-based labeling. For each of these regexes, if a report contained a match, it was given the corresponding label.

For each of the four classification tasks, the classifier with the highest macro-averaged *F*_1_ score on the validation set was selected. This selection criterion is simple but does not account for high performance occurring by chance (23). Once the four best performing classifiers were selected (one for each classification task), their generalization performance was estimated on the held-out test set.

## Results

Table 1 shows the selected features, grouped by category, for each of the four classification tasks. The first classification task (classification with respect to ER label), involves a much lower number of retained features than the other three. These other three classification tasks also share a similar distribution of features in each category.

**Table 1:** Numbers and types of selected features for each of the four classification tasks.

	**Total extracted**	**Selected for ER**	**Selected for PR**	**Selected for HER2 (IHC)**	**Selected for *HER2* (ISH)**
Number of regex matches	3,344	2	22	21	9
Location(s) of regex match(es) in the report	15,120	1	50	49	51
Numeric values	78	0	3	1	5
Word counts	Not fixed	0	4	1	1
Jaccard (lexicographic) similarities with reference sentences	468	2	3	7	3
Boolean combinations of features	1,168	4	8	16	6
Logistic regressions of features	105	1	5	1	5
Total number of features	~20,000	10	95	96	80

Except for the ER biomarker, all classifiers seem to share a common pattern in which locations of the regex matches are the most selected features. On average, relevant numeric values and word counts were the least selected types of features, as shown in Table 1. The reason is that relevant numeric values cannot be extracted for all reports, because they are not always provided, or not in a clean way. Word counts are not very useful, most probably because of the bilingual nature of this work. For instance, even if one word was perfectly able to classify Dutch reports, it was probably absent in the French reports, making it on average, only a half-perfect predictor. The bilingual corpus was considered using regexes grouping translations of the same concept. As previously mentioned, the location of the concepts in the report is also very important. In previous work, Pironet et al. observed that Jaccard similarities were superior to single words for classifying single report sentences (24). This observation can be repeated here for the classification of whole reports. Finally, manual design of feature combinations is a time-consuming task but seems to be of added value. As shown in Table 2, for classification of reports into ER categories, the created feature combinations seem to be so well-performing that only 10 features were necessary to complete this task (out of 20,000). This observation is also true for the other three classification tasks, for which 95, 96, and 80 features were selected. Moreover, this small dimensionality obtained thanks to the manual design of features allows to better represent and interpret the classifiers, and thus gain more insight into the data.

**Table 2:** Results of the four classification tasks.

**Marker**	**Number of features**	**Best classifier**	**Macro-averaged *F*_**1**_ score on the validation set (number of reports)**	**Macro-averaged *F*_**1**_ score on the test set (number of reports)**
ER	10	SVM (linear kernel)	0.90 (*N* = 273)	0.91 (*N* = 258)
PR	95	Random forest	0.93 (*N* = 285)	0.92 (*N* = 265)
HER2 (IHC)	96	Logistic regression	0.92 (*N* = 250)	0.92 (*N* = 265)
*HER2* (ISH)	80	SVM (linear kernel)	0.89 (*N* = 244)	0.89 (*N* = 274)

Table 2 also presents the selected classifier for each of the four classification tasks, i.e., the one with the highest macro-averaged *F*_1_ score on the validation set. The last column shows the performance of the selected classifiers on the held-out test set, for which the macro-averaged *F*_1_ scores ranged from 0.89 to 0.92. These results are well above the performance of the rule-based classifiers, which ranged between 0.39 and 0.54. These results are also close to 1, which represents a perfect classifier.

Furthermore, it can be observed that the macro-averaged *F*_1_ scores for the held-out test set are on par with those for the validation set, suggesting that there was no overfit and that the classifiers can safely be used on new data.

## Discussion

This study was designed to evaluate the possibility of automatically extracting the status of the 3 main breast cancer biomarkers (ER, PR, and HER2) from the contents of pathology reports written in two different languages, and coming from 82 different providers, using conventional machine learning models.

After testing different classifiers, the best performing ones achieved macro-averaged *F*_1_ scores ranging from 0.89 to 0.92 on the held-out test sets, which is on par with best efforts in the literature (6, 7, 11, 12). The reported *F*_1_ scores in the literature range between 0.87 and 1, but use only three possible labels for HER2, whereas five are used in the present work.

The classifiers were tested once more on another, more recent dataset of 524 breast cancer pathology reports randomly selected from the incidence year 2017 (the classifiers were trained and evaluated on data from the incidence year 2014). The macro-averaged *F*_1_ scores were evaluated on this new, independent dataset and are as follows:

- ER: 0.81
- PR: 0.84
- HER2 (IHC): 0.89
- *HER2* (ISH): 0.76.

The performances of the classifiers are, as could be expected, lower on this new dataset, but still much higher than for the rule-based classifiers. The lower performance could be attributed to some limited overfitting, to a difference in the distribution of the data between 2014 and 2017, or most probably to a mixture of both. Specifically, the lowest score for *HER2* (ISH) is caused by the difficult categories “Carried out but no result” and “Equivocal,” for reasons discussed in section Causes of Classification Errors. The corresponding accuracy of this fourth classifier is 91%.

In the present work, an effort was put on the manual engineering of relevant features. The classifiers produced in this work included remarkably low numbers of selected features, suggesting that manual feature engineering is powerful enough to justify the time it takes. Manual feature engineering also made it possible to deal with reports in two languages without having to develop one classifier for each language. This latter approach would have required more training work, using half the available data. Moreover, it would not have been able to process reports containing both languages, which happens if an additional analysis is performed in a laboratory using a different language than the one of the first laboratory. Nevertheless, this well-performing algorithm has a few limits to be aware of, but which have been analyzed thoroughly.

### Limits

#### Causes of Classification Errors

Several challenges were encountered and can explain some of the misclassifications. First, errors can be caused by reports presenting results of IHC tests for other biomarkers but written in a very similar fashion as IHC test results for ER, PR, or HER2. Second is the presence of the results of HER2 IHC controls in a report, as shown in Figure 1: a positive control has result 3+, a negative one has result 0 or 1+ (8). These additionally present results complicate the extraction of the true conclusive result. A third cause of errors is when a report contains results related to multiple tumors, typically bilateral breast tumors, in which the classifier has trouble making a final decision. Such reports can easily be detected and discarded in the BCR database, but at present cannot reliably be automatically processed. Reports describing multiple breast tumors represent <1% of all reports available at the BCR (Macq et al., manuscript in preparation).

Some additional difficulties were specifically encountered for the extraction of the *HER2* (ISH) status. First, classification into the “Carried out but no result” category was error-prone because many reports state that an ISH test is ongoing, and the results of this ISH test are reported further in the text. An example is given in Figure 1. In addition, the category “Equivocal” does not seem to be interpreted in the same way by all pathologists in 2014. While the guidelines state in which conditions a sample must be reported as equivocal (3, 5, 8, 9), it was observed that, in such conditions, many pathologists reported the sample as negative.

#### Manual Annotation

Another limitation of the present work is that annotators had various levels of clinical expertise regarding the diagnosis of breast cancer, even though all annotators received training and were supported for this specific task. In addition, reports are written in Dutch or in French, meaning that, in some cases, annotators had to process complex medical information in their non-native language. As in other studies (6), human error is inevitable during manual annotation. Some incorrect manual labels were identified during development and have been corrected. To a posteriori quantify the difficulty of the annotation task, two of the authors (NVD and LVW) independently annotated 524 additional reports from the incidence year 2017. The inter-rater agreement, also measured using the macro-averaged *F*_1_ scores, ranged between 0.92 and 0.96 depending on the classification task. Given enough training and time, annotators can thus perform very well. On the other hand, because of the large heterogeneity in how the reports are written, rule-based classifiers did not provide good results.

#### Performance of All Classifiers

As mentioned in section Classifiers and Training, many different classifiers were built on the training set and evaluated on the validation set. For each classification task, the best classifier was selected as the one with the highest macro-averaged *F*_1_ score on the validation set and is reported in Table 1. Given no statistical test was performed to compare classifiers with one another, it cannot be guaranteed that the classifiers selected in Table 1 significantly outperform the other ones. Despite this limitation, the selected classifiers still performed very well on two held-out test datasets, as previously described.

### Perspectives

#### Merging of the Report-Level Labels at Tumor-Level

The developed method has already been applied to a large subset of the BCR database, providing receptor status for more than 200,000 breast cancer reports (incidence period 2008–2017), allowing to perform more focused real-world population-based studies of quality of care for breast cancer diagnostic, prognostic assessment as well as clinical management (10). For some specific projects, the next step after obtaining report-level labels for each biomarker is to merge this information to obtain a single label at tumor-level for each biomarker (in particular if there are discordant results for the same biomarker which may be studied twice on two different samples such as a biopsy and surgical excision). With the help of an expert pathologist, we have built a set of merging rules for this purpose and are currently evaluating it.

#### State of the Art NLP Tools

Clinical NLP tools can be separated into three categories: rule-based (7, 13), conventional machine learning (11, 12), and deep learning (6). The present study used a range of conventional machine learning classifiers, and different classifiers were selected for the different classification tasks. This observation emphasizes that trying many different classifiers is often the key to success with machine learning. Now that this first work has proven that it is possible to correctly extract information from free-text pathology reports at the BCR, deep learning certainly represents the next step. We plan to try state-of-the-art techniques in text classification, such as deep learning which has proved to be highly efficient in labeling medical cancer-related free-text (6, 25). Deep learning and word embeddings also offer the possibility to process texts in different languages (6), which is appealing for our two-language setting.

#### Relevance to Biomarkers for Other Cancer Types

Similar classifiers could be relevant for many other categories of biomarkers which are needed for diagnostic, prognostic, or therapeutic purposes, such as prognostic scores [Gleason score for prostate tumors (14, 26)], viral tumor status [Human Papilloma Virus for cervical or oropharynx cancers (2, 24)], protein expression [targeted inhibitors for ALK-positive lung cancers (27) or immune checkpoint inhibitors in tumors expressing PDL1], gene mutations (targeted inhibitors for *BRAF* V600-mutated melanoma). In all these new projects, we plan to improve upon the presented methodology, for instance using transfer learning to build upon the previous work rather than re-starting from scratch.

## Conclusion

This work presents a method for automatic extraction of breast receptor biomarker status from free-text pathology reports, using machine-learning classifiers. The classifiers showed results as good as other studies on the same topic with macro-averaged *F*_1_ scores ranging from 0.89 to 0.92. The methodology was developed at a national level and can extract this relevant information from reports written in two different languages by a very large number of providers. We have shown that, overall, using machine learning tools contributes to automatization of data extraction, increasing the availability and quality of clinically relevant data at the BCR, and potentially at other registries processing breast cancer pathology reports in Dutch and/or French. Automatically extraction of data within pathology reports by NLP allows registries to rapidly increase their dataset with up-to-date biomarkers without increasing the workload of hospital data managers. The availability of an extended and regularly updated dataset for a cancer registry enables in-depth studies to provide international studies with population-scale data in a real-world setting and to guide national policy regarding personalized medicine.

## Data Availability Statement

The datasets presented in this article are not readily available because the dataset contains confidential health-related data that cannot be shared. Requests to access the datasets should be directed to info@kankerregister.org.

## Author Contributions

AP, HP, HD, LW, JM, LV, and NV contributed to conception and design of the study. HP, JM, and NV supervised the manual data annotation process. KH and JM provided the database. AP performed the analyses. AP, HP, and TT wrote the manuscript. All authors contributed to manuscript revision, read, and approved the submitted version.

## Conflict of Interest

AP is now an employee of AARDEX Group, whose customers include several pharmaceutical companies. The remaining authors declare that the research was conducted in the absence of any commercial or financial relationships that could be construed as a potential conflict of interest.

## Publisher's Note

All claims expressed in this article are solely those of the authors and do not necessarily represent those of their affiliated organizations, or those of the publisher, the editors and the reviewers. Any product that may be evaluated in this article, or claim that may be made by its manufacturer, is not guaranteed or endorsed by the publisher.

## Footnotes

^1^https://kankerregister.org/media/docs/Exempleprotocoles-version2020.txt and https://kankerregister.org/media/docs/Voorbeeldprotocols%E2%80%93versie2020.txt

^2^The commonly used name HER2 will be used throughout this manuscript.

## Appendix A: Definition of the Macro-Averaged ***F***_**1**_ Score

### Definition of the ***F***_**1**_ Score in a Two-Label Setting

For a two-label classification problem, the reference and predicted labels are either 0 or 1. Each report thus belongs to one (and only one) of the following categories:

- True negatives, for which the reference label is 0 and the predicted label is 0;
- False positives, for which the reference label is 0 and the predicted label is 1;
- False negatives, for which the reference label is 1 and the predicted label is 0;
- True positives, for which the reference label is 1 and the predicted label is 1.

The *F*_1_ score is defined as follows:

$$\begin{array}{l} F_{1}=\frac{2N_{TP}}{2N_{TP}+N_{FP}+N_{FN}}, \end{array}$$

where *N*_*TP*_ is the number of true positives, *N*_*FP*_, the number of false positives, and *N*_*FN*_, the number of false negatives.

### Definition of the Macro-Averaged ***F***_**1**_ Score in a Multi-Label Setting

This section explains how to generalize the *F*_1_ score in a setting with *k* possible labels, denoted *C*_1_, …, *C*_*k*_. To apply the *F*_1_ score to a multi-label setting with *k* possible labels, the *k*-label classification problem must be conceptually transformed into *k* two-label classification problems. For each of these *k* two-label classification problems, the two reference and predicted labels are either *C*_*i*_ (corresponding to 1) or not *C*_*i*_ (corresponding to 0), with *i* = 0, …, *k*. The following categories are then introduced:

- True negatives, for which the reference label is not *C*_*i*_ and the predicted label is not *C*_*i*_;
- False positives, for which the reference label is not *C*_*i*_ and the predicted label is *C*_*i*_;
- False negatives, for which the reference label is *C*_*i*_ and the predicted label is not *C*_*i*_;
- True positives, for which the reference label is *C*_*i*_ and the predicted label is *C*_*i*_.

For example, if the possible labels are “Positive,” “Negative” and “Unknown,” there are three two-label classification problems. For the first two-label classification problem, the possible labels are “Positive” and “Not positive,” which contains all reports with the labels “Negative” or “Unknown.” For the second two-label classification problem, the possible labels are “Negative” and “Not negative,” *etc*.

Using the definition of the *F*_1_ score for the two-label setting, *k* different *F*_1_ scores can be computed: one for each of the *k* two-label classification problems. The average of these *k* different *F*_1_ scores is called the macro-averaged *F*_1_ scores and measures the performance of the *k*-label classifier.

### Appendix B: Feature Normalization

All features, discrete and continuous, were normalized so that their mean was equal to zero, and their variance was equal to one. Let μ(*f*) be the mean of the feature *f* and σ(*f*), its standard deviation, both computed on the training set only. The normalized feature is:

$$\begin{array}{l} f^{\prime }=\frac{f-\mu \left(f\right)}{\sigma \left(f\right)}. \end{array}$$

Features for which σ(*f*) was too small were excluded, as part of the feature selection process. The normalization factors, μ(*f*) and σ(*f*), were stored so that they could be used for normalizing the validation and test set features.
